# *Utricularia lihengiae* (Lentibulariaceae), a new species from Northwest Yunnan, China

**DOI:** 10.3897/phytokeys.177.63346

**Published:** 2021-04-26

**Authors:** Zhuo Cheng, Qiong Fang, Fei Wang, Chun-Lin Long

**Affiliations:** 1 College of Life and Environmental Sciences, Minzu University of China, Beijing 100081, China Minzu University of China Beijing China; 2 Key Laboratory of Ethnomedicine (Minzu University of China), Ministry of Education, Beijing 100081, China Ministry of Education Beijing China; 3 Key Laboratory of Ecology and Environment in Minority Areas (Minzu University of China), National Ethnic Affairs Commission, Beijing 10 0081, China National Ethnic Affairs Commission Beijing China

**Keywords:** Bladderwort, insectivorous plant, taxonomy, Yunnan Province

## Abstract

*Utricularia lihengiae*, a new species from the Dulongjiang region of northwest Yunnan, China, is here described and illustrated. The new species belongs to the section Oligocista and is similar to *U. bifida* L. and *U. scandens* Benj., from which it can be easily distinguished by the dark purple stripe on the corolla. The new species also differs in its shorter inflorescence and the shape of the calyx lobes.

## Introduction

Lentibulariaceae is a monophyletic family composed of three carnivorous genera: *Utricularia*Linnaeus (1753: 18), *Pinguicula*Linnaeus (1753: 17) and *Genlisea*Saint-Hilaire (1833: 428) (Taylor 1989; Fleischmann et al. 2010). *Utricularia* spp., commonly called bladderworts, is the largest genus in this family (Taylor 1974). For a long time, *Utricularia* has attracted a great deal of interest in its peculiar morphology and carnivorous characteristics (Taylor 1974; Li 1988; Fleischmann et al. 2010). *Utricularia* plants are typically small but complex in structure, with vegetative organs that are highly variable. It is difficult to dissect and observe *Utricularia* specimens after they are dried, providing difficulties in the accurate identification and classification of taxa within the genus (Albert et al. 1992).

In his monograph, Taylor (1989) recognized a total of 214 species in *Utricularia* worldwide and classified them into two subgenera and 35 sections. Since then, some sixty species of *Utricularia* have been published from different parts of the world. Currently, about 274 species of *Utricularia* have been described (Fleischmann 2012, 2015; Delprete 2014; Kumar et al. 2018; Hong et al. 2021).

*Utricularia* is the largest carnivorous plant genus and also one of the most widely distributed. The genus is known to occur on every continent except for Antarctica (Taylor 1989). The vast majority of species are found in tropical and sub-tropical regions, particularly where conditions are seasonally wet, with high or very high annual rainfall.

Currently, 25 species of *Utricularia* have been reported in China. Most of them are mainly distributed in the provinces to the south of the Yangtze River. With 13 species, Yunnan has the highest diversity of *Utricularia* in China (Li 1990, 2007).

**Figure 1.**
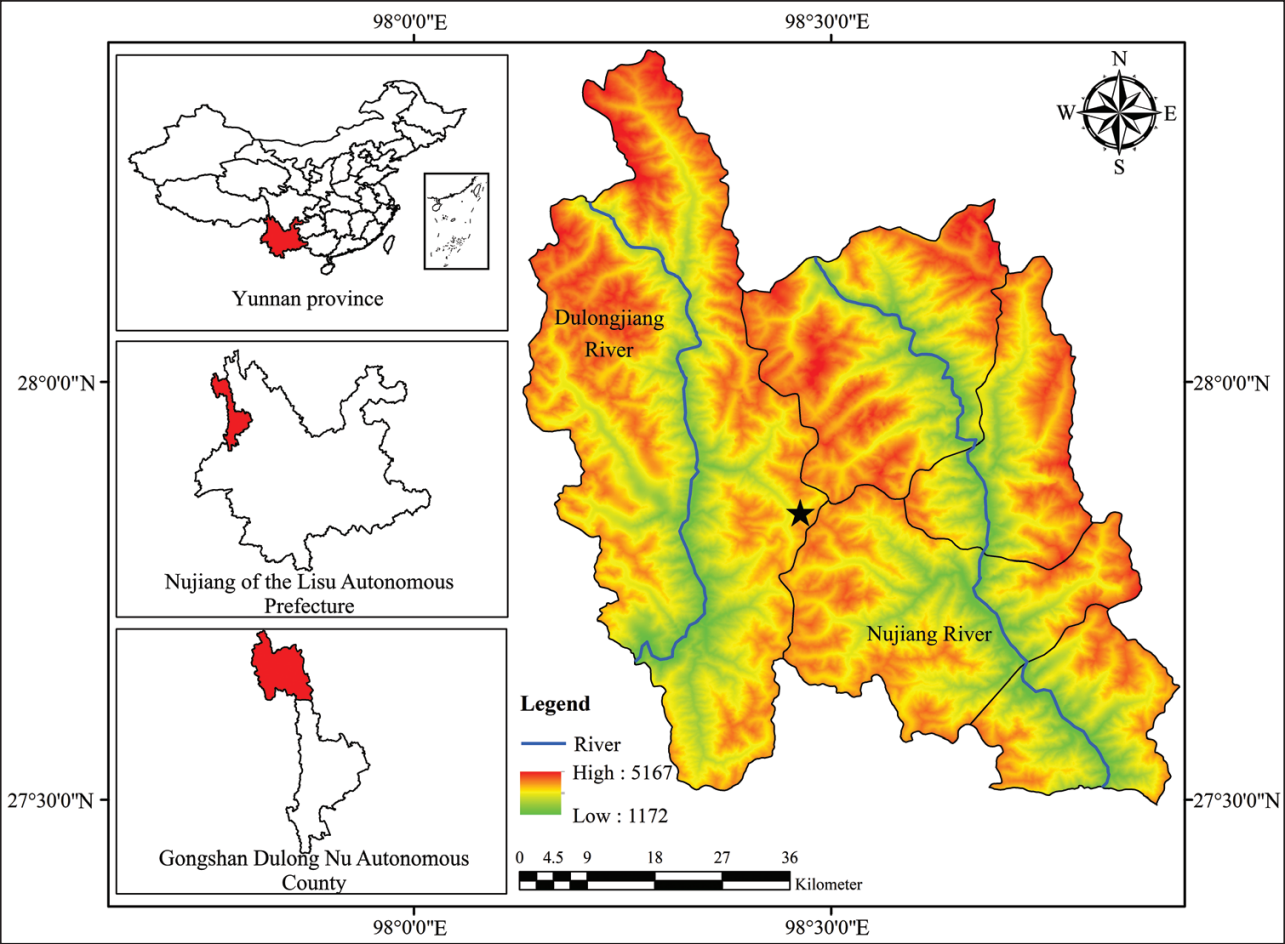
Distribution of *Utricularia lihengiae*.

In August 2019, the authors visited Dulongjiang in Northwest Yunnan, an isolated area of the Eastern Himalayas, to conduct a survey on traditionally used plants and biodiversity associated traditional knowledge (Figure 1). The Dulongjiang region is located in the core area of Gaoligongshan National Nature Reserve, adjacent to Chayu County (Tibetan Autonomous Region, China) to the north and Kachin State (Myanmar) to the west and south. Dulongjiang region has among the highest levels of flora and faunal biodiversity in China (Li et al. 2011). During the survey work, a species of *Utricularia* was discovered growing in moss at the north entrance of the Dulongjiang tunnel. With dark purple stripes on the corolla, the species is very distinctive. After reviewing Taylor’s monograph (1989), it can be determined that this species belongs to the section OligocistaTaylor (1989: 305) as explained in Taxonomic Notes below. In November, the authors visited the Dulongjiang area again to collect specimens for further investigations. After detailed examinations, the taxon is here described as a new species, *Utricularia lihengiae*.

## Material and methods

This study was based on field observations and detailed examinations of herbarium specimens. Herbarium specimens collected from northwest Yunnan were deposited at the Herbarium, Kunming Institute of Botany, Chinese Academy of Sciences (**KUN**) (Thiers 2020). A comparative study of herbarium collections (PE, KUN, IBK, WUK, and IBSC) revealed an undescribed taxon in the genus *Utricularia*. Dried specimens were examined using a dissecting microscope (XTL-Iab, Beijing Keyi Electro-optical Instrument Factory). Detailed observation and measurement of the collected individuals were conducted covering the rhizoid, stolon, leaf, traps, calyx lobe, bracts, flowers, and spurs. Conservation status was assessed applying the IUCN Red List categories and criteria, version 3.1 (IUCN 2012). For comparison, the unknown species and related specimens in herbaria, the monographs of Taylor (1989) and the contribution of Li (1990, 2007) were also referenced.

## Taxonomic treatment

### Utricularia lihengiae

Taxon classificationPlantaeLamialesLentibulariaceae

C. L. Long & Z. Cheng sp. nov.

2262D1C1-205D-57EA-A840-FADA4B29ED3A

urn:lsid:ipni.org:names:77216662-1

**Figure 2.**
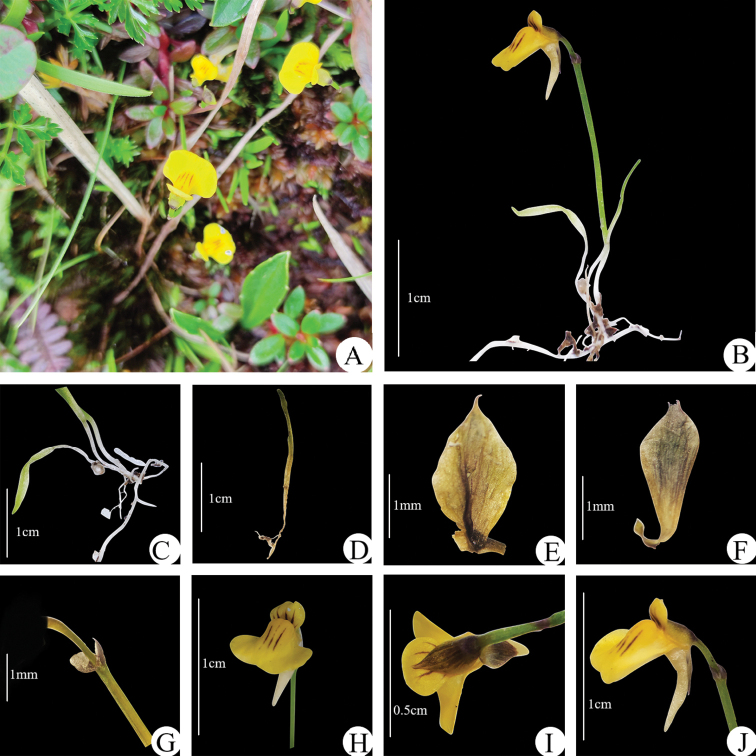
*Utricularia lihengiae* (**A–J**) **A** habitat **B** whole plant **C** traps and laminar leaves **D** leaf **E** calyx upper lobe **F** calyx lower lobe **G** bracts **H–J** frontal, dorsal and lateral view of the flower (**A–J** Photos by Z. Cheng.).

“李恒挖耳草” (Li Heng Wa Er Cao) Figure 2 , Table 1

#### Diagnosis.

*U. lihengiae* is similar to *U. bifida* L. (1753: 18), but differs by the inflorescences 2–4 cm long (vs. mostly 10–20 cm long in *U. bifida*), calyx upper and lower with apex acuminate (vs. calyx upper lobe, apex obtuse, calyx lower lobe, apex rounded or very shortly bifid in *U. bifida*), 3–5 dark purple stripes on the upper corolla lip, 3 dark purple stripes on lower corolla lip (vs. absent in the upper and lower corolla lip in *U. bifida*); *U. lihengiae* is similar to *U. scandens* Benj. (1847: 309), but differs by the smaller inflorescence 2–4 cm long (vs. mostly 15–35 cm long in *U. scandens*), peduncle of *U. lihengiae* is erect (vs. peduncle usually twining in *U. scandens*), calyx lower lobe with apex acuminate (vs. calyx lower lobe with apex rounded or very shortly bifid in *U. scandens*), upper calyx lobe of *U. lihengiae* is shorter than upper corolla lip (vs. upper calyx lobe longer than upper corolla lip in *U. scandens*), 3–5 dark purple stripes on the upper corolla lip, 3 dark purple stripes on lower corolla lip (vs. absent in the upper and lower corolla lip in *U. scandens*).

#### Type.

China, Yunnan Province, Nujiang Lisu Autonomous Prefecture, Gongshan Dulong and Nu Autonomous County, Dulongjiang Township, 2844 m a.s.l., 27°50'36"N, 98°27'48"E, 3 September, 2019, *Chun Lin Long & Zhuo Cheng DXH066*, (holotype: KUN!; isotype: KUN!).

**Table 1.** Morphological comparison among *Utricularia lihengiae*, *U. bifida*, and *U. scandens*.

Character	*U. lihengiae*	*U. bifida*	*U. scandens*
Inflorescence	erect, 2–4 cm long	erect, mostly 10–20 cm long	erect or twining, mostly 15–35 cm long
Peduncle	peduncle erect	peduncle usually erect	peduncle usually twining
Calyx upper lobe	apex acuminate	apex obtuse	apex acuminate
Calyx lower lobe	apex acuminate	apex rounded or very shortly bifid	apex shortly and acutely bifid
Calyx upper lobe and upper corolla lip	upper calyx lobe shorter than upper	upper calyx lobe shorter than upper	upper calyx lobe longer than upper
	corolla lip	corolla lip	corolla lip
Upper corolla lip	3–5 dark purple stripes	stripes absent, upper lip slightly constricted below the middle	stripes absent, upper lip constricted below the middle
Lower corolla lip	3 dark purple stripes	stripes absent, the base with a prominent rounded swelling	stripes absent, the base distinctly swollen, with 2 or 4 rounded, longitudinal ridges, the apex rounded or obscurely 2–3 crenate

#### Description.

Small annual herbs. Rhizoids few, capillary, 0.5 cm to 2 cm long, 0.15–0.3 mm thick, with numerous short papillose branches. Stolons few, capillary, branched, up to 4 cm long, ca. 0.2 mm thick, the internodes mostly 2–4 mm long. Leaves few, from the stolon nodes, petiolate, the lamina narrowly linear, with apex rounded or subacute, 1–nerved, 0.5–1 mm wide, total length up to 1.5 cm. Traps rather few on the stolon internodes and leaves, globose, shortly stalked, 0.5–1 mm long, the mouth basal with 2 simple, subulate, reflexed, dorsal appendages and with a ± distinct rounded swelling on the ventral side of the mouth or on the adjacent distal part of the stalk. Inflorescence erect, solitary, simple or rarely sparsely branched, 2–4 cm long; peduncle terete, glabrous, 0.2–0.4 mm thick. Scales few, similar to the bracts. Bracts basifixed, ovate, with apex obtuse to acute, 1–2 mm long, 1–5 nerved. Bracteoles subulate, with apex acute, much shorter than the bract. Flowers 1–2, the raceme axis elongate; pedicels spreading at anthesis, decurved in fruit, capillary, broadly winged, 2–6 mm long. Calyx lobes slightly unequal, broadly ovate, 1–3 mm long, the upper lobe with apex obtuse to acute, the lower lobe slightly smaller with apex obtuse to acute. Corolla yellow, 3–7 mm long; upper lip slightly constricted below the middle, the superior part oblong or oblong–obovate, with apex rounded, bearing 3–5 dark purple stripes on the upper corolla lip, radial, the inferior part broadly ovate–deltoid; lower lip limb galeate, approximately circular, the base with a prominent rounded swelling, the apical margin rounded; palate margin ciliate; 3 dark purple stripes on the lower corolla lip, parallel; spur subulate, with apex acute, curved, about as long as and widely diverging from the lower lip. Filaments straight, 1 mm long, the anther thecae distinct. Ovary ovoid, dorsiventrally compressed; style distinct; stigma lower lip semicircular, the upper lip very short or ± obsolete. Capsule broadly ellipsoid, dorsiventrally compressed, 2.5–3 mm long, the wall uniformly membranous, dehiscing by dorsal and ventral longitudinal slits. Seeds obliquely obovoid, the major end with apex subtruncate, 0.4–0.5 mm long, the testa cells elongate with anticlinal boundaries much raised and longitudinally striate, somewhat sinuate, the periclinal walls tabular, conspicuously longitudinally striate.

#### Distribution and habitat.

The only known locality of this taxon is in Dulongjiang Township, Gongshan Dulong and Nu Autonomous County, Northwest Yunnan, China. The site is located in an open area in a primeval forest dominated by Fagaceae, Magnoliaceae and Ericaceae. The observed population is very small, with fewer than 80 plants growing in the moss amongst damp grass on the roadside, accompanied by the moss *Polytrichum commune*Hedwig (1801: 88), as well as *Vaccinium chaetothrix*Sleumer (1941: 432), and *Acorus tatarinowii*Schott (1859: 101). The elevation is 2800–2900 metres above sea level. The climate here is rainy and humid, with rain falling for most of the year.

#### Phenology.

Flowering and fruiting occurs from August to November.

#### Etymology.

Named in honor of Prof. Li Heng, a Chinese botanist who has made significant contributions to the knowledge of the flora of Dulongjiang region.

#### Conservation status.

This species has not been recorded or described so far, and there is only one known site in Dulongjiang region, which is relatively unknown to botanists. In addition, *Utricularia lihengiae* is very small and has a short flowering period, making it easily overlooked. This species satisfies the IUCN 3.1 Red List CR (Critically Endangered) Criteria B1ab(ii,iii)+2ab(i,ii,iii) (IUCN 2012), which has an EOO (Extent of occurrence) < 100 km^2^ and AOO (Area of occupancy) < 10 km^2^, it may be classified as “critically endangered” (CR). The distribution site of *Utricularia lihengiae* is next to the road, which is at great risk of human disturbance and extreme weather, such as tourist activities, road building, grazing and landslides. Additionally, regional management in pursuit of economic development is likely to pose a threat through trampling and pollution of soil and water, causing negative impacts to the small and fragile habitat.

#### Taxonomic notes.

The new species belongs to Utricularia section Oligocista due to the following characters: traps globose, the mouth basal with 2 simple subulate dorsal appendages and leaves linear to obovate (Taylor 1989). There were five species belonging to Utricularia section Oligocista in China prior to the discovery of *U. lihengiae*. From the perspective of geographical distribution, *U. bifida* and *U. scandens* are both distributed in Yunnan, *U. scandens*, is mainly distributed in northwestern Yunnan, whereas *U. bifida* is mainly found in south Yunnan. According to the key to the species of *Utricularia* occurring in China (Li 2007), the morphology of *U. lihengiae* is similar to *U. bifida* and *U. scandens* in the yellow corolla. However, it can be clearly distinguished by the dark purple stripes of the corolla, shorter inflorescence and by the shape of the calyx lobes. A comparative summary of the characters that differentiate these three taxa is presented in Table 1.

#### Additional specimens examined.

China, Yunnan Province, Nujiang Lisu Autonomous Prefecture, Gongshan Dulong and Nu Autonomous County, Dulongjiang Township, 2844 m a.s.l., 27°50'36"N, 98°27'48"E, 3 September 2019, *Chun Lin Long & Zhuo Cheng DXH066*, *Chun Lin Long & Zhuo Cheng DXH067*, *Chun Lin Long & Zhuo Cheng DXH068*, *Chun Lin Long & Zhuo Cheng DXH069*, *Chun Lin Long & Zhuo Cheng DXH070* (KUN!).

#### Specimens of *Utricularia bifida* examined.

CHINA. Guangdong: Renhua County, 16 November 1973, *C.J. Huang & Y.T. Zhang 077* (PE); Deqing County, 5 August 1958, *Y.G. Liu 01303* (PE), 2 August 1930, *J.L. Zuo 22497* (IBK), 6 July 1958, *X.G. Li 202078* (IBK). Guangxi: Yongning District, 6 July 1984, *Z.Y. Li 10984* (PE); Lingui County, 5 September 1997, *G.Z. Li 16229* (PE), 11 October 1958, *Y.K Li 402165* (IBK), 19 October 1948, *S.G. Li 200104* (IBK). Yunnan, 2 June 1939, *M.K. Li 1698* (WUK). Jiangxi, 19 September 1963, *J.S. Yue et al. 3983* (IBSC), 26 June 1932, *Y. Jiang 9967* (IBSC).

### Key to species of Utricularia section Oligocista occurring in China

**Table:** 

1	Corolla yellow	**2**
–	Corolla violet, mauve, lilac, or white	**4**
2	Peduncle erect; pedicel strongly recurved in fruit; upper calyx lobe shorter than upper corolla lip, apex obtuse	**3**
–	Peduncle twining to erect; pedicel erect in fruit; upper calyx lobe longer than upper corolla lip, apex shortly acuminate	***U. scandens***
3	Dark purple stripes in upper corolla lip and lower corolla lip	***U. bifida***
–	Stripes absent in upper corolla lip and lower corolla lip	***U. lihengiae***
4	Peduncle twining; pedicel strongly deflexed in fruit	***U. foveolata***
–	Peduncle erect; pedicel erect to spreading in fruit	**5**
5	Fruiting pedicel as long as or shorter than fruiting calyx; calyx lobes suborbicular in fruit; seeds globose, with isodiametric reticulations; leaf blade 2.5–4.5 cm × 1.5–6 mm	***U. uliginosa***
–	Fruiting pedicel much longer than fruiting calyx; calyx lobes ovate in fruit; seeds ovoid to ellipsoid, with elongate reticulations; leaf blade 0.4–2 cm × 0.8–3 mm	***U. graminifolia***

## Supplementary Material

XML Treatment for Utricularia lihengiae
